# Timing of endoscopy in patients with cirrhosis and acute variceal bleeding: a single-center retrospective study

**DOI:** 10.1186/s12876-023-02766-8

**Published:** 2023-06-26

**Authors:** Mengyuan Peng, Zhaohui Bai, Deli Zou, Shixue Xu, Chunmei Wang, Metin Başaranoğlu, Cyriac Abby Philips, Xiaozhong Guo, Xiaodong Shao, Xingshun Qi

**Affiliations:** 1 Department of Gastroenterology, General Hospital of Northern Theater Command (formerly General Hospital of Shenyang Military Area), No. 83 Wenhua Road, Shenyang, Liaoning Province 110840 China; 2 Postgraduate College, Jinzhou Medical University, Jinzhou, P.R. China; 3 Postgraduate College, Shenyang Pharmaceutical University, Shenyang, P.R. China; 4 Department of Internal Medicine, Bezmialem Vakıf University Faculty of Medicine, İstanbul, Turkey; 5 Clinical and Translational Hepatology & Monarch Liver Laboratory, The Liver Institute, Center of Excellence in Gastrointestinal Sciences, Rajagiri Hospital, Aluva, Kerala India

**Keywords:** Acute variceal bleeding, Liver cirrhosis, Endoscopy, Five-day failure to control bleeding, In-hospital mortality

## Abstract

**Background:**

The optimal timing of endoscopy in liver cirrhosis with acute variceal bleeding (AVB) remains controversial in current guidelines and studies.

**Methods:**

Consecutive patients with liver cirrhosis and AVB were screened. The timing of endoscopy was calculated from the last presentation of AVB or the admission to endoscopy. Early endoscopy was defined as the interval < 12 h, < 24 h, or < 48 h. A 1:1 propensity score matching (PSM) analysis was performed. Five-day failure to control bleeding and in-hospital mortality were evaluated.

**Results:**

Overall, 534 patients were included. When the timing of endoscopy was calculated from the last presentation of AVB, PSM analysis demonstrated that the rate of 5-day failure to control bleeding was significantly higher in early endoscopy group defined as < 48 h (9.7% versus 2.4%, P = 0.009), but not < 12 h (8.7% versus 6.5%, P = 1.000) or < 24 h (13.4% versus 6.2%, P = 0.091), and that the in-hospital mortality was not significantly different between early and delayed endoscopy groups (< 12 h: 6.5% versus 4.3%, P = 1.000; <24 h: 4.1% versus 3.1%, P = 1.000; <48 h: 3.0% versus 2.4%, P = 1.000). When the timing of endoscopy was calculated from the admission, PSM analyses did not demonstrate any significant difference in the rate of 5-day failure to control bleeding (< 12 h: 4.8% versus 12.7%, P = 0.205; <24 h: 5.2% versus 7.7%, P = 0.355; <48 h: 4.5% versus 6.0%, P = 0.501) or in-hospital mortality (< 12 h: 4.8% versus 4.8%, P = 1.000; <24 h: 3.9% versus 2.6%, P = 0.750; <48 h: 2.0% versus 2.5%, P = 1.000) between early and delayed endoscopy groups.

**Conclusion:**

Our study could not support any significant association of timing of endoscopy with cirrhotic patients with AVB.

**Supplementary Information:**

The online version contains supplementary material available at 10.1186/s12876-023-02766-8.

## Introduction

Acute variceal bleeding (AVB) is a fatal complication in liver cirrhosis. The incidence of variceal bleeding is about 10-15% per year and 6-week mortality is 10–20% [1–3]. Current practice guidelines regarding the management of AVB mainly include blood volume restitution, antibiotic prophylaxis, vasoactive drugs, endoscopy, transjugular intrahepatic portosystemic shunt (TIPS), and salvage balloon tamponade [2, 4].

Despite endoscopy is the mainstay choice of treatment for AVB in patients with cirrhosis, the timing of endoscopy remains controversial in current guidelines. The Baveno VI consensus, the American Association for the Study of Liver Diseases (AASLD) practice guideline, the Belgian guideline, and the European Society of Gastrointestinal Endoscopy (ESGE) guideline recommend endoscopy within 12 h of presentation of AVB [2, 3, 5, 6]. The UK guideline recommends that endoscopy should be performed as soon as possible after resuscitation in patients with severe and unstable AVB, and within 24 h after admission in the remaining patients [7]. The Chinese guideline recommends that endoscopy should ideally be performed within 12-24 h since the presentation of AVB [8].

Previous studies regarding the timing of endoscopy in liver cirrhosis with AVB had contradictory conclusions. A retrospective cohort study, including 311 patients with cirrhosis and AVB, found that delayed endoscopy (> 15 h) was an independent risk factor for in-hospital mortality [9]. However, another retrospective cohort study found that the timing of endoscopy might not influence the 6-week mortality in patients with cirrhosis and AVB [10]. For this reason, we further performed an updated meta-analysis, showing that early endoscopy might be beneficial for overall survival of patients with cirrhosis and AVB, but not significantly associated with control of bleeding [11]. However, multiple factors, including the definition regarding timing of endoscopy, presentation of AVB, hemodynamic status, and severity of liver disease, could not be sufficiently addressed or adjusted in our meta-analysis.

More recently, a large randomized controlled trial (RCT) did not demonstrate any significant difference in 30-day mortality between patients with upper gastrointestinal bleeding treated with urgent endoscopy (< 6 h) and early endoscopy (6-24 h) [12]. However, these findings may not be appropriate for the management of AVB, because most patients included in this RCT were non-cirrhotic (91.8%), and the source of bleeding was mostly non-variceal (82.9%) [12].

Herein, we conducted a retrospective study to further shed light on whether early endoscopy was beneficial for patients with cirrhosis and AVB, especially by adjusting for the definitions regarding timing of endoscopy and early endoscopy, manifestation of AVB, and severity of liver disease.

## Methods

### Study design

Patients with liver cirrhosis and AVB were screened from our retrospective database of 982 patients with liver cirrhosis and acute upper gastrointestinal bleeding (AUGIB) who were consecutively admitted to our hospital between January 2010 and June 2014 [13, 14] and our prospective database of 346 patients with liver cirrhosis and AUGIB who were consecutively admitted to our department between December 2014 and January 2022 [15, 16]. Age and comorbidities were not limited.

Exclusion criteria were as follows: (1) patients did not undergo endoscopy or those with contraindications for endoscopy; (2) patients underwent endoscopy at other hospitals or emergency department or outpatient clinics of our hospital; (3) endoscopy was performed beyond 5 days after the last episode of AUGIB; (4) the timing of endoscopy was ambiguous according to the medical records; and (5) the source of AUGIB was non-variceal or could not be accurately identified according to the medical records or endoscopic reports.

The study protocol has been approved by the Medical Ethical Committee of the General Hospital of Northern Theater Command with an approval number (Y [2022] 019) and performed according to the Declaration of Helsinki. Written informed consents were waived due to the retrospective nature of this study.

### Data collection

Primary data extracted included age, gender, clinical manifestations, etiology of liver disease, presence of hepatocellular carcinoma, systolic blood pressure, heart rate, hemoglobin, white blood cell, platelet count, albumin, alanine aminotransferase, blood urea nitrogen, serum creatinine, sodium, and prothrombin time (PT) at admission. Active variceal bleeding on endoscopy, source of variceal bleeding, endoscopic variceal therapy, surgery or interventional treatment, Child-Pugh score and class, and model for end-stage liver disease (MELD) score were also recorded.

### Outcomes

Outcomes of interest included 5-day failure to control bleeding and in-hospital death.

### Definitions

Timing of endoscopy was calculated according to the interval from the last presentation of AUGIB or the admission to endoscopy. Accordingly, eligible patients were divided into early and delayed endoscopy groups according to three different intervals, including < 12 h versus ≥ 12 h, < 24 h versus ≥ 24 h, and < 48 h versus ≥ 48 h.

AUGIB was defined as hematemesis or melena within 120 h before admission [2]. Variceal bleeding was defined as: (1) the presence of active bleeding from varices on endoscopy; (2) signs of recent bleeding, such as white nipple; or (3) variceal bleeding would also be considered, if varices were the only lesion in the stomach, and blood was found in the stomach or endoscopy was performed 24 h after bleeding [3]. Five-day failure to control bleeding was defined as the presence of any of the following within 5 days after endoscopy: (1) vomiting fresh blood or aspirating more than 100 ml fresh blood for patients with naso-gastric tube placement after 2 h of endoscopy; (2) reduction of 3 g/L hemoglobin without transfusion; or (3) death [2].

### Management of AUGIB

Generally, the management of AUGIB is in accordance with the current practice guideline, which primarily includes fluid resuscitation, blood transfusion, pharmacological treatment, and endoscopic treatment [1, 8]. Red blood cell transfusion would be given, if patients had a hemoglobin level of < 60-80 g/L, or they had active bleeding and were hemodynamically unstable. Pharmacological treatment included intravenous vasoactive drugs (terlipressin, somatostatin, or octreotide) and high-dose proton pump inhibitors. Timing of endoscopy was decided at the discretion of attending physicians according to the patients’ age, consciousness, comorbidities, and hemodynamics. Endoscopic treatment was performed by experienced endoscopists. Endoscopic variceal therapy includes endoscopic variceal ligation, sclerotherapy, and injection of tissue adhesive. Endoscopic variceal ligation was primarily employed for the treatment of acute esophageal variceal bleeding, sclerotherapy was considered when ligation was technically difficult or active variceal bleeding was observed on endoscopy, and injection of tissue adhesive was primarily used for the treatment of acute gastric variceal bleeding. Repeat endoscopy was often recommended 2–4 weeks after the first endoscopic variceal therapy, with additional endoscopic variceal treatment(s), if necessary. If endoscopy failed to control bleeding, patients would undergo surgery or interventional treatment.

### Statistical analyses

Continuous variables were presented as mean ± standard deviation and median (range), and categorical variables were presented as frequency (percentage). The non-parametric Mann-Whitney U test was used for continuous variables, and the chi-square test and Fisher’s exact test were used for categorical variables to explore the difference between early and delayed endoscopy groups. Logistic and Cox regression analyses were performed to identify whether early endoscopy was an independent predictor of 5-day failure to control bleeding or in-hospital death. Odds ratios (ORs) and hazard ratios (HRs) with 95% confidence intervals (CIs) were calculated. A 1:1 propensity score matching (PSM) analysis was performed by using a matching tolerance of 0.02 and greedy-matching algorithm without replacement to compare the rate of 5-day failure to control bleeding and in-hospital mortality between early and delayed endoscopy groups. After PSM, the comparability of baseline characteristics between the groups was re-evaluated. Matching factors included age, gender, systolic blood pressure < 90mmHg, heart rate > 100 beats per minute, PT, Child-Pugh score, MELD score, hematemesis at admission, active variceal bleeding on endoscopy, and endoscopic variceal therapy. A two-tailed P < 0.05 was considered statistically significant. All statistical analyses were performed with IBM SPSS 26.0 (IBM Corp, Armonk, NY, USA).

## Results

### Patients

**Fig. 1:**
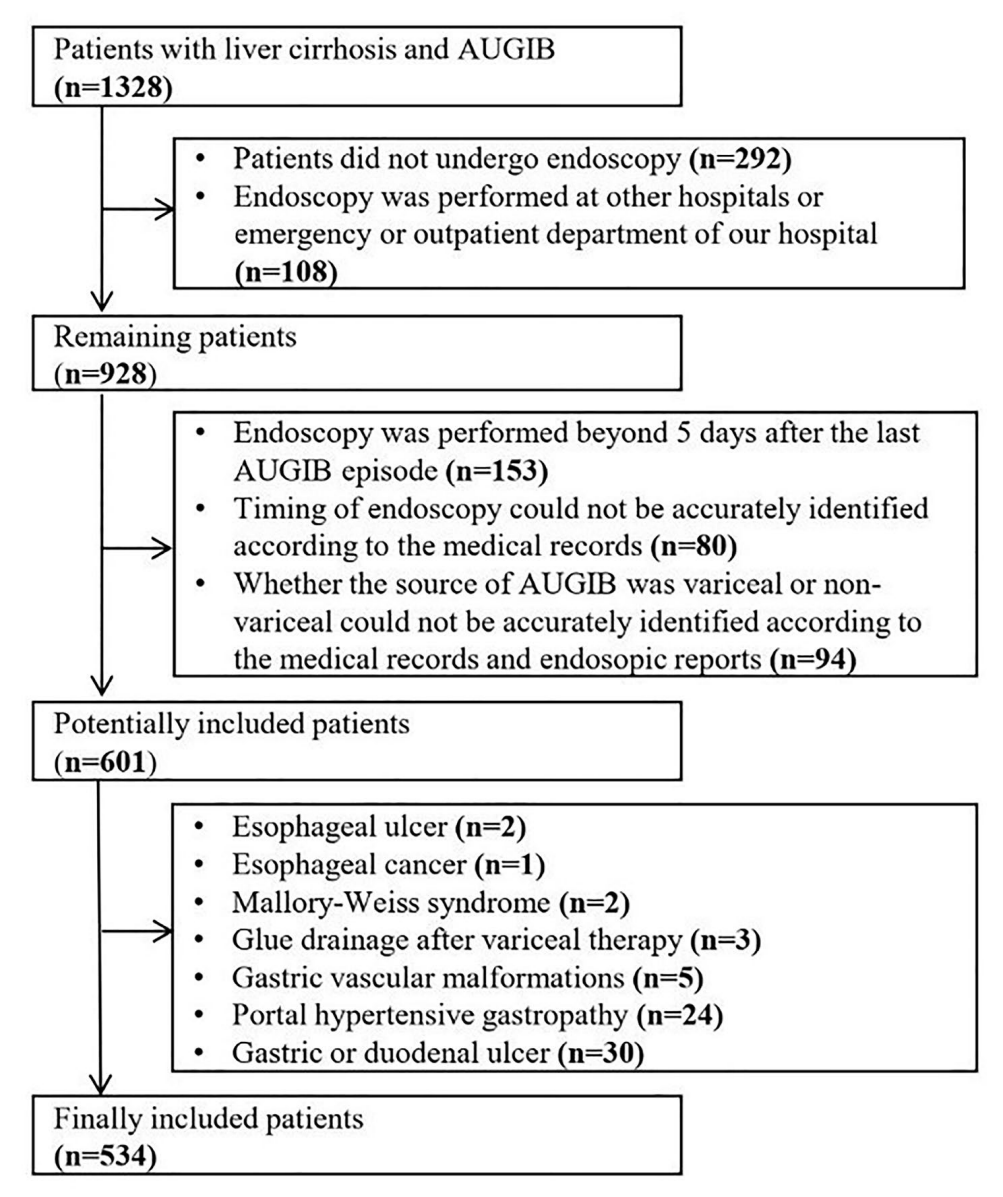
Flowchart of patient selection

Overall, 534 patients with cirrhosis and AVB were included (Fig. 1). Patient characteristics were shown in Table 1. The median age was 55.66 years (range: 6.28–92.31 years), and 376 (70.4%) patients were male. Hematemesis at admission in 332 (62.2%) patients. Most patients had Child-Pugh class B/C (333/498, 66.9%). Median MELD score was 10.51 (range: 6.43–38.01). Active bleeding was observed under endoscopy in 39 (7.4%) patients. The rate of 5-day failure to control bleeding was 5.1% (n = 27). The in-hospital mortality was 2.4% (n = 13). The causes of death included massive gastrointestinal bleeding (n = 8), end-stage liver disease with multiple organ failure (n = 4), and cardiogenic shock (n = 1).

**Table 1:** Characteristics of included patients

Variables	No. Pts	Mean ± SD or Median (Range) or Frequency (Percentage)
**Age (years)**	534	55.66 (6.28–92.31) 55.78 ± 11.61
**Male**	534	376 (70.4%)
**Etiology of underlying liver diseases**	534	
Hepatitis B virus		222 (41.6%)
Hepatitis C virus		47 (8.8%)
Alcohol abuse		150 (28.1%)
**Hepatocellular carcinoma**	534	48 (9.0%)
**Hematemesis**	534	332 (62.2%)
**Hemodynamics**		
Heart rate (beats per minute)	534	80.00 (44.00-148.00) 83.36 ± 13.24
Heart rate > 100 beats per minute	534	54 (10.1%)
Systolic blood pressure (mmHg)	533	115.00 (75.00-176.00) 116.07 ± 17.59
Systolic blood pressure < 90mmHg	533	18 (3.4%)
**Laboratory tests**		
Hemoglobin (g/L)	533	73.00 (23.00-158.00) 77.53 ± 22.51
White blood cell (10^12^/L)	533	4.40 (1.00-46.10) 5.45 ± 3.98
Platelet count (10^9^/L)	533	72.00 (15.00-457.00) 87.44 ± 58.82
Total bilirubin (µmol/L)	529	19.90 (3.30-187.40) 25.01 ± 18.35
Albumin (g/L)	524	30.50 (10.00-50.70) 30.57 ± 6.53
Alanine aminotransferase (U/L)	527	22.73 (5.00-1064.00) 32.70 ± 58.03
Blood urea nitrogen (mmol/L)	512	8.03 (1.54–42.83) 8.95 ± 4.79
Serum creatinine (µmol/L)	510	62.00 (25.00-715.00) 69.30 ± 45.29
Sodium (mmol/L)	524	138.5 (109.2-160.10) 138.44 ± 4.22
Prothrombin time (seconds)	510	16.00 (10.50–55.00) 16.85 ± 4.00
**Child-Pugh score**	498	7.00 (5.00–13.00) 7.47 ± 1.82
**Child-Pugh class A/B + C**	498	165 (33.1%)/333 (66.9%)
**MELD score**	502	10.51 (6.43–38.01) 11.92 ± 4.61
**Source of variceal bleeding**	519^*^	
Esophageal varices (%)		313 (60.3%)
Gastric varices (%)		77 (14.8%)
Esophageal and gastric varices (%)		129 (24.9%)
**Active variceal bleeding on endoscopy**	528^#^	39 (7.4%)
**Endoscopic variceal therapy**	534	496 (92.9%)
**Surgery or interventional treatment**	534	3 (0.6%)
**Rate of 5-day failure to control bleeding**	534	27 (5.1%)
**In-hospital mortality**	534	13 (2.4%)

**Abbreviations**: MELD, model for end-stage liver disease. Notes: * Source of variceal bleeding was unclear in 15 patients because of missing medical records or poor visual fields under endoscopy; ^**#**^ Active variceal bleeding on endoscopy cannot be identified in 6 patients because of missing endoscopic reports.

### Time to endoscopy according to the interval from the last presentation of AVB to endoscopy

***< 12 h versus ≥ 12 h.*** In the overall-analysis, early endoscopy group had significantly higher proportions of hepatitis C virus (HCV) infection and systolic blood pressure < 90 mmHg and white blood cell than delayed endoscopy group (Supplementary Table 1). Early endoscopy group had significantly higher rate of 5-day failure to control bleeding and in-hospital mortality than delayed endoscopy group (Table 2).

**Table 2:** Outcomes according to the timing of endoscopy defined as the interval from the last presentation of AVB to endoscopy

***Overall analysis***	**< 12h** **(N = 55)**	**≥ 12h** **(N = 434)**	**P value**	**< 24h** **(N = 114)**	**≥ 24h** **(N = 357)**	**P value**	**< 48h** **(N = 226)**	**≥ 48h** **(N = 242)**	**P value**
Rate of 5-day failure to control bleeding	6 (10.9%)	14 (3.2%)	***0.007***	15 (13.2%)	8 (2.2%)	***< 0.001***	20 (8.8%)	5 (2.1%)	***0.001***
In-hospital mortality	5 (9.1%)	6 (1.4%)	***< 0.001***	6 (5.3%)	6 (1.7%)	***0.035***	8 (3.5%)	5 (2.1%)	0.332
***PSM analysis***	**< 12h** **(N = 46)**	**≥ 12h** **(N = 46)**	**P value**	**< 24h** **(N = 97)**	**≥ 24h** **(N = 97)**	**P value**	**< 48h** **(N = 165)**	**≥ 48h** **(N = 165)**	**P value**
Rate of 5-day failure to control bleeding	4 (8.7%)	3 (6.5%)	1.000	13 (13.4%)	6 (6.2%)	0.091	16 (9.7%)	4 (2.4%)	***0.009***
In-hospital mortality	3 (6.5%)	2 (4.3%)	1.000	4 (4.1%)	3 (3.1%)	1.000	5 (3.0%)	4 (2.4%)	1.000

**Abbreviations:** AVB, acute variceal bleeding; PSM, propensity score matching

Multivariate logistic regression analysis demonstrated that the interval from last presentation of AVB to endoscopy < 12 h was not significantly associated with 5-day failure to control bleeding (OR = 2.889, 95% CI: 0.912–9.151; P = 0.071). Multivariate Cox regression analysis showed that the interval from last presentation of AVB to endoscopy < 12 h was not significantly associated with in-hospital death (HR = 2.828, 95% CI: 0.706–11.320, P = 0.142).

In the PSM analysis, 46 patients were matched to each group (Supplementary Table 2). There was no significant difference in rate of 5-day failure to control bleeding or in-hospital mortality between the two groups (Table 2).

***< 24 h versus ≥ 24 h.*** In the overall-analysis, early endoscopy group was significantly older and had higher proportions of HCV infection, hematemesis, systolic blood pressure < 90 mmHg, Child-Pugh class B/C, and active variceal bleeding on endoscopy and white blood cell than delayed endoscopy group (Supplementary Table 3). Early endoscopy group had significantly higher rate of 5-day failure to control bleeding and in-hospital mortality than delayed endoscopy group (Table 2).

Multivariate logistic regression analysis demonstrated that the interval from last presentation of AVB to endoscopy < 24 h was significantly associated with a higher rate of 5-day failure to control bleeding (OR = 6.065, 95% CI: 2.336–15.749; P < 0.001). Multivariate Cox regression analysis showed that the interval from last presentation of AVB to endoscopy < 24 h was not significantly associated with in-hospital death (HR = 1.400, 95% CI: 0.403–4.860, P = 0.597).

In the PSM analysis, 97 patients were matched to each group (Supplementary Table 4). There was no significant difference in rate of 5-day failure to control bleeding or in-hospital mortality between the two groups (Table 2).

***< 48 h versus ≥ 48 h.*** In the overall-analysis, early endoscopy group was significantly older and had significantly higher proportions of HCV infection, hematemesis, systolic blood pressure < 90 mmHg, Child-Pugh class B/C, and active variceal bleeding on endoscopy, white blood cell, and blood urea nitrogen than delayed endoscopy group (Supplementary Table 5). Early endoscopy group had a significantly higher rate of 5-day failure to control bleeding than delayed endoscopy group, but a statistically similar in-hospital mortality (Table 2).

Multivariate logistic regression analysis demonstrated that the interval from last presentation of AVB to endoscopy < 48 h was significantly associated with a higher rate of 5-day failure to control bleeding (OR = 4.171, 95% CI: 1.486–11.708; P = 0.007). Cox regression analysis showed that the interval from last presentation of AVB to endoscopy < 48 h was not significantly associated with in-hospital death (HR = 0.808, 95% CI: 0.237–2.750, P = 0.733).

In the PSM analysis, 165 patients were matched to each group (Supplementary Table 6). Early endoscopy group had a significantly higher rate of 5-day failure to control bleeding than delayed endoscopy group, but a statistically similar in-hospital mortality (Table 2).

### Time to endoscopy according to the interval from the admission to endoscopy

***< 12 h versus ≥ 12 h.*** In the overall-analysis, early endoscopy group was significantly older and had significantly higher proportions of HCV infection, Child-Pugh class B/C, and active variceal bleeding on endoscopy, white blood cell, total bilirubin, blood urea nitrogen, Child-Pugh score, and MELD score than delayed endoscopy group (Supplementary Table 7). There was no significant difference in rate of 5-day failure to control bleeding or in-hospital mortality between the two groups (Table 3).

**Table 3:** Outcomes according to the timing of endoscopy defined as the interval from the admission to endoscopy

***Overall analysis***	**< 12h** **(N = 75)**	**≥ 12h** **(N = 453)**	**P value**	**< 24h** **(N = 175)**	**≥ 24h** **(N = 349)**	**P value**	**< 48h** **(N = 289)**	**≥ 48h** **(N = 236)**	**P value**
Rate of 5-day failure to control bleeding	5 (6.7%)	21 (4.6%)	0.451	10 (5.7%)	17 (4.9%)	0.680	13 (4.5%)	13 (5.5%)	0.596
In-hospital mortality	4 (5.3%)	8 (1.8%)	0.055	7 (4.0%)	6 (1.7%)	0.113	8 (2.8%)	5 (2.1%)	0.634
***PSM analysis***	**< 12h** **(N = 63)**	**≥ 12h** **(N = 63)**	**P value**	**< 24h** **(N = 155)**	**≥ 24h** **(N = 155)**	**P value**	**< 48h** **(N = 199)**	**≥ 48h** **(N = 199)**	**P value**
Rate of 5-day failure to control bleeding	3 (4.8%)	8 (12.7%)	0.205	8 (5.2%)	12 (7.7%)	0.355	9 (4.5%)	12 (6.0%)	0.501
In-hospital mortality	3 (4.8%)	3 (4.8%)	1.000	6 (3.9%)	4 (2.6%)	0.750	4 (2.0%)	5 (2.5%)	1.000

**Abbreviations:** PSM, propensity score matching

Multivariate logistic regression analysis demonstrated that the interval from admission to endoscopy < 12 h was not significantly associated with 5-day failure to control bleeding (OR = 0.873, 95% CI: 0.243–3.144; P = 0.836). Multivariate Cox regression analysis showed that the interval from admission to endoscopy < 12 h was not significantly associated with in-hospital death (HR = 1.981, 95% CI: 0.467–8.406, P = 0.354).

In the PSM analysis, 63 patients were matched to each group (Supplementary Table 8). There was no significant difference in rate of 5-day failure to control bleeding or in-hospital mortality between the two groups (Table 3).

***< 24 h versus ≥ 24 h.*** In the overall-analysis, early endoscopy group was significantly older and had significantly higher proportion of Child-Pugh class B/C and Child-Pugh score than delayed endoscopy group (Supplementary Table 9). There was no significant difference in rate of 5-day failure to control bleeding or in-hospital mortality between the two groups (Table 3).

Multivariate logistic regression analysis demonstrated that the interval from admission to endoscopy < 24 h was not significantly associated with 5-day failure to control bleeding (OR = 0.867, 95% CI: 0.353–2.132; P = 0.756). Multivariate Cox regression analysis showed that the interval from admission to endoscopy < 24 h was not significantly associated with in-hospital death (HR = 2.554, 95% CI: 0.747–8.732, P = 0.135).

In the PSM analysis, 155 patients were matched to each group (Supplementary Table 10). There was no significant difference in rate of 5-day failure to control bleeding or in-hospital mortality between the two groups (Table 3).

***< 48 h versus ≥ 48 h.*** In the overall-analysis, early endoscopy group was significantly older than delayed endoscopy group (Supplementary Table 11). There was no significant difference in rate of 5-day failure to control bleeding or in-hospital mortality between the two groups (Table 3).

Multivariate logistic regression analysis demonstrated that the interval from admission to endoscopy < 48 h was not significantly associated with 5-day failure to control bleeding (OR = 0.643, 95% CI: 0.275–1.506; P = 0.309). Multivariate Cox regression analysis showed that the interval from admission to endoscopy < 48 h was not significantly associated with in-hospital death (HR = 1.513, 95% CI: 0.416–5.499, P = 0.529).

In the PSM analysis, 199 patients were matched to each group (Supplementary Table 12). There was no significant difference in rate of 5-day failure to control bleeding or in-hospital mortality between the two groups (Table 3).

### Subgroup analyses

Subgroup analyses were performed according to the manifestations of AUGIB (hematemesis versus non-hematemesis) and severity of liver diseases (Child-Pugh class A versus Child-Pugh class B/C). The results were shown in Supplementary Table 13 and Supplementary Table 14.

## Discussion

Based on the data obtained from our center, the benefit of early endoscopy on the outcomes of patients with liver cirrhosis and AVB could not be supported. It seems to be contrary to the traditional concept that early endoscopy could achieve more rapid hemostasis and hence better outcomes. Notably, early endoscopy may influence basic resuscitation, leading to ischemic complications, and shorten the duration of action of vasoactive drugs or antibiotics before endoscopic treatment for acute gastrointestinal bleeding [17]. Additionally, there were a significantly larger number of patients with active bleeding in early endoscopy group. Thus, a large amount of blood and contents in the non-fasting stomach limits the visual field under endoscopy and masks the primary source of bleeding, thereby increasing the technical difficulty as well as risk of aspiration or perforation [18]. By comparison, delayed endoscopy may be safer and provides clearer visual field, especially after portal pressure has been sufficiently decreased by the use of vasoactive drugs [10].

Our finding may be influenced by the definition regarding time to endoscopy, the manifestation of AVB, or the severity of liver dysfunction. First, until now, any standard definition regarding time to endoscopy has not been given by any practice guideline yet [2, 3, 6, 19, 20]. Indeed, the definitions regarding time to endoscopy are heterogeneous among previous studies. By contrast, in the present study, both of two major definitions, of which one refers to the interval from the last presentation of gastrointestinal bleeding to endoscopy [21], and another refers to the interval from the admission to endoscopy [9, 22], have been employed. It is true that the number and percentage of patients assigned to early endoscopy group were different (55 [10.3%] in < 12 h endoscopy group according to the first definition versus 75 [14.0%] in < 12 h endoscopy group according to the second definition). On the other hand, the definitions regarding early endoscopy are also different among previous studies, including < 6h [23], < 12h [21, 22] and < 24h [24]. Accordingly, the results of overall analyses based on the interval from the last presentation of AVB to endoscopy are also heterogeneous that < 12 h and < 24 h endoscopy group, but not < 48 h endoscopy group, had significantly higher in-hospital mortality than delayed endoscopy group.

Second, patients with cirrhosis and AVB who presented with hematemesis at admission may have worse prognosis than those who presented with melena [14]. The results of our subgroup analyses based on the interval from the last presentation of AVB to endoscopy demonstrated that early endoscopy was significantly associated with a higher risk of 5-day failure to control bleeding in patients with hematemesis, but such an association remains in patients with non-hematemesis. By contrast, a previous cohort study by Chen et al. found that 6-week rebleeding rate and mortality were lower in patients with hematemesis who underwent early endoscopy than those who underwent delayed endoscopy, but not significantly different between the two groups in patients without hematemesis [22]. Such a difference between current and previous studies may be because all of the patients included in the Chen’s study had active AVB, but active bleeding was observed endoscopically in only 7.4% of our patients. Rapid control of active bleeding by early endoscopy is beneficial for preventing from an injury to the liver and other organs and achieving better outcomes. It can be speculated that patients with hematemesis without active bleeding will benefit more from delayed endoscopy after sufficient medical therapy as compared to early endoscopy.

Third, the severity of liver cirrhosis affects the prognosis of AVB [25]. It has been confirmed that MELD score is an independent predictor for the prognosis of liver cirrhosis with AVB [26, 27]. Huh et al. found that early endoscopy, which was defined as the interval from the last presentation of gastrointestinal bleeding to endoscopy ≤ 12 h, increased the risk of 6-week rebleeding and death in the low-risk (MELD score ≤ 17) group, but the timing of endoscopy was not associated with the prognosis in the high-risk (MELD score > 17) group [21]. By comparison, according to the results of our subgroup and PSM analyses, the association between timing of endoscopy and outcomes was not influenced by Child-Pugh class or score. Thus, the optimal timing of endoscopy may not be dependent upon the severity of liver cirrhosis.

Our study has several limitations as follows. First, our study was retrospective. The selection of emergency endoscopy often depends on the patients’ conditions, physicians’ decisions, and availability of endoscopists, which leads to a considerable selection bias. Specifically, patients with massive hematemesis who were not effectively treated by drugs were more prone to early endoscopy, those with hemodynamic instability would undergo delayed endoscopy after basic resuscitation, and those admitted during off-hours might undergo delayed endoscopy. Although all procedures were performed by experienced endoscopists, it may be different in judging the source of gastrointestinal bleeding and treating AVB under endoscopy among endoscopists, which influences the prognosis of patients. In addition, endoscopic findings in the stomach were retrospectively derived from the patients’ medical records alone, but they seemed to be insufficient to evaluate whether gastric contents had influenced the efficacy of gastric variceal treatment under endoscopy. Second, our study excluded patients who had undergone endoscopy at their local hospitals and outpatient and emergency departments, those who were not suitable for endoscopy, as well as those who died before endoscopy. Such patients should be more severe and critical. Third, this was a single-center study, which should be validated by a multi-center study. In addition, our study had a relatively small number of death events, which might be unpowered to achieve statistically significant results between early and delayed endoscopy groups.

In conclusion, our study did not confirm an association of timing of endoscopy with risk of 5-day failure to control bleeding or in-hospital death in patients with cirrhosis and AVB. Endoscopy after adequate medical therapy may be more effective than urgent endoscopy. RCTs with strict eligibility criteria and optimal definitions regarding timing of endoscopy are needed to confirm the effect of the timing of endoscopy on prognosis of patients with cirrhosis and AVB in the future.

## Electronic supplementary material

Below is the link to the electronic supplementary material.

Supplementary Material 1

## Data Availability

The dataset data used to support the findings of this study are available from the corresponding author at email address upon request.

## Abbreviations

AASLD: American Association for the Study of Liver Diseases
AUGIB: Acute upper gastrointestinal bleeding
AVB: Acute variceal bleeding
ESGE: European Society of Gastrointestinal Endoscopy
HCV: Hepatitis C virus
MELD: Model for End-stage Liver Disease
PSM: Propensity score matching
PT: Prothrombin time
RCT: Randomized controlled trial
TIPS: Trans-jugular intrahepatic portosystemic shunt

## References

[1] Garcia-Tsao G, Bosch J (2010). Management of varices and variceal hemorrhage in cirrhosis. N Engl J Med.

[2] de Franchis R (2015). Expanding consensus in portal hypertension: report of the Baveno VI Consensus Workshop: stratifying risk and individualizing care for portal hypertension. J Hepatol.

[3] Garcia-Tsao G, Abraldes JG, Berzigotti A, Bosch J (2017). Portal hypertensive bleeding in cirrhosis: risk stratification, diagnosis, and management: 2016 practice guidance by the American Association for the study of liver diseases. Hepatology.

[4] Hwang JH, Shergill AK, Acosta RD, Chandrasekhara V, Chathadi KV, Decker GA (2014). The role of endoscopy in the management of variceal hemorrhage. Gastrointest Endosc.

[5] Colle I, Wilmer A, Le Moine O, Debruyne R, Delwaide J, Dhondt E (2011). Upper gastrointestinal tract bleeding management: belgian guidelines for adults and children. Acta Gastroenterol Belg.

[6] Karstensen JG, Ebigbo A, Bhat P, Dinis-Ribeiro M, Gralnek I, Guy C (2020). Endoscopic treatment of variceal upper gastrointestinal bleeding: european Society of Gastrointestinal Endoscopy (ESGE) Cascade Guideline. Endoscopy Int open.

[7] Tripathi D, Stanley AJ, Hayes PC, Patch D, Millson C, Mehrzad H (2015). U.K. guidelines on the management of variceal haemorrhage in cirrhotic patients. Gut.

[8] Xu X, Ding H, Jia J, Wei L, Duan Z, Linghu E (2016). Guidelines for the diagnosis and treatment of esophageal and gastric variceal bleeding in cirrhotic portal hypertension. J Clin Hepatol.

[9] Hsu YC, Chung CS, Tseng CH, Lin TL, Liou JM, Wu MS (2009). Delayed endoscopy as a risk factor for in-hospital mortality in cirrhotic patients with acute variceal hemorrhage. J Gastroenterol Hepatol.

[10] Yoo JJ, Chang Y, Cho EJ, Moon JE, Kim SG, Kim YS (2018). Timing of upper gastrointestinal endoscopy does not influence short-term outcomes in patients with acute variceal bleeding. World J Gastroenterol.

[11] Bai Z, Wang R, Cheng G, Ma D, Ibrahim M, Chawla S (2021). Outcomes of early versus delayed endoscopy in cirrhotic patients with acute variceal bleeding: a systematic review with meta-analysis. Eur J Gastroenterol Hepatol.

[12] Lau JYW, Yu Y, Tang RSY, Chan HCH, Yip HC, Chan SM (2020). Timing of Endoscopy for Acute Upper Gastrointestinal Bleeding. N Engl J Med.

[13] An Y, Bai Z, Xu X, Guo X (2020). No benefit of hemostatic drugs on Acute Upper gastrointestinal bleeding in cirrhosis. Biomed Res Int.

[14] Li Y, Li H, Zhu Q, Tsochatzis E, Wang R, Guo X (2019). Effect of acute upper gastrointestinal bleeding manifestations at admission on the in-hospital outcomes of liver cirrhosis: hematemesis versus melena without hematemesis. Eur J Gastroenterol Hepatol.

[15] Yi F, Guo X, Wang L, Xu X, An Y, Tang Y (2021). Impact of spontaneous splenorenal shunt on liver volume and long-term survival of liver cirrhosis. J Gastroenterol Hepatol.

[16] Yin Y, Li Y, Shao L, Yuan S, Liu B, Lin S (2021). Effect of Body Mass Index on the prognosis of liver cirrhosis. Front Nutr.

[17] Baradarian R, Ramdhaney S, Chapalamadugu R, Skoczylas L, Wang K, Rivilis S (2004). Early intensive resuscitation of patients with upper gastrointestinal bleeding decreases mortality. Am J Gastroenterol.

[18] Merola E, Michielan A, de Pretis G (2021). Optimal timing of endoscopy for acute upper gastrointestinal bleeding: a systematic review and meta-analysis. Intern Emerg Med.

[19] Reiberger T, Püspök A, Schoder M, Baumann-Durchschein F, Bucsics T, Datz C (2017). Austrian consensus guidelines on the management and treatment of portal hypertension (Billroth III). Wien Klin Wochenschr.

[20] EASL Clinical Practice Guidelines for the management of patients with decompensated cirrhosis (2018). J Hepatol.

[21] Huh CW, Kim JS, Jung DH, Yang JD, Nam SW, Kwon JH, et al. Optimal endoscopy timing according to the severity of underlying liver disease in patients with acute variceal bleeding. Dig Liver Dis. 2019;51(7):993–8. 10.1016/j.dld.2019.01.013 10.1016/j.dld.2019.01.013 30803858

[22] Chen PH, Chen WC, Hou MC, Liu TT, Chang CJ, Liao WC (2012). Delayed endoscopy increases re-bleeding and mortality in patients with hematemesis and active esophageal variceal bleeding: a cohort study. J Hepatol.

[23] Badave RR, Tantry V, Gopal S, Shenoy S, Shetty A (2017). Very early (< 6&nbsp;h) endoscopic therapy affects the outcome in acute variceal bleeding: a retrospective study from tertiary care hospital in south india. J Clin Exp Hepatol.

[24] Wang Z, Gao F (2018). Analysis of timing and influencing factors of endoscopic diagnosis and treatment for cirrhotic patients with esophageal variceal bleeding. Chin J Gastroenterol.

[25] Peng Y, Qi X, Guo X (2016). Child-Pugh versus MELD score for the assessment of prognosis in liver cirrhosis: a systematic review and Meta-analysis of observational studies. Medicine.

[26] Bambha K, Kim WR, Pedersen R, Bida JP, Kremers WK, Kamath PS (2008). Predictors of early re-bleeding and mortality after acute variceal haemorrhage in patients with cirrhosis. Gut.

[27] Reverter E, Tandon P, Augustin S, Turon F, Casu S, Bastiampillai R (2014). A MELD-based model to determine risk of mortality among patients with acute variceal bleeding. Gastroenterology.

