# Supplementary material for: Timing of endoscopy in patients with cirrhosis and acute variceal bleeding: a single-center retrospective study
Source: BMC Gastroenterol. 2023 Jun 26;23:219. doi: 10.1186/s12876-023-02766-8 (PMC10291747; doi:10.1186/s12876-023-02766-8)
Supplement: Supplementary file 1 — Supplementary Material 1 [file 12876_2023_2766_MOESM1_ESM.docx]

| **Supplementary table 1. Difference between patients undergoing endoscopy <12h and ≥12h according to the interval from the last presentation of AVB to endoscopy** | | | | | | |
| --- | --- | --- | --- | --- | --- | --- |
| **Variables** | **<12h group** | |  | **≥12h group** | | **P value** |
|  | **No. Pts** | **Median (Range) or Frequency (Percentage)** |  | **No. Pts** | **Median (Range) or Frequency (Percentage)** |  |
| **Age (years)** | 55 | 59.76 (27.49-88.73) |  | 434 | 54.92 (6.28-92.31) | 0.081 |
| **Male** | 55 | 39 (70.9%) |  | 434 | 304 (70.0%) | 0.895 |
| **Etiology of underlying liver diseases** |  |  |  |  |  |  |
| Hepatitis B virus | 55 | 17 (30.9%) |  | 434 | 187 (43.1%) | 0.084 |
| Hepatitis C virus | 55 | 9 (16.4%) |  | 434 | 32 (7.4%) | ***0.023*** |
| Alcohol abuse | 55 | 21 (38.2%) |  | 434 | 117 (27.0%) | 0.081 |
| **Hematemesis** | 55 | 34 (61.8%) |  | 434 | 263 (60.6%) | 0.862 |
| **Hepatocellular carcinoma** | 55 | 7 (12.7%) |  | 434 | 33 (7.6%) | 0.191 |
| **Hemodynamics** |  |  |  |  |  |  |
| Heart rate (beats per minute) | 55 | 80.00 (59.00-120.00) |  | 434 | 80.00 (44.00-148.00) | 0.598 |
| Heart rate >100 beats per minute | 55 | 7 (12.7%) |  | 433 | 43 (9.9%) | 0.516 |
| Systolic blood pressure (mmHg) | 55 | 114.00 (75.00-154.00) |  | 433 | 116.00 (75.00-176.00) | 0.150 |
| Systolic blood pressure <90mmHg | 55 | 6 (10.9%) |  | 433 | 11 (2.5%) | ***0.001*** |
| **Laboratory tests** |  |  |  |  |  |  |
| Hemoglobin (g/L) | 55 | 72.00 (47.00-157.00) |  | 433 | 74.00 (23.00-158.00) | 0.936 |
| White blood cell (10^12^/L) | 55 | 5.30 (1.30-46.10) |  | 433 | 4.20 (1.00-26.00) | ***0.008*** |
| Platelet count (10^9^/L) | 55 | 70.00 (22.00-457.00) |  | 433 | 71.00 (15.00-435.00) | 0.710 |
| Total bilirubin (μmol/L) | 54 | 21.15 (5.50-81.40) |  | 430 | 19.75 (3.30-107.00) | 0.194 |
| Albumin (g/L) | 53 | 29.80 (17.30-49.30) |  | 427 | 30.90 (10.00-50.70) | 0.283 |
| Alanine aminotransferase (U/L) | 54 | 24.50 (7.00-1064.00) |  | 428 | 22.00 (5.0-617.00) | 0.577 |
| Blood urea nitrogen (mmol/L) | 52 | 8.36 (3.04-28.25) |  | 416 | 7.87 (2.03-42.83) | 0.683 |
| Serum creatinine (μmol/L) | 52 | 61.00 (28.00-327.00) |  | 414 | 61.43 (25.00-715.00) | 0.651 |
| Sodium (mmol/L) | 54 | 138.9 (109.20-159.00) |  | 426 | 138.55 (123.00-160.10) | 0.756 |
| Prothrombin time (seconds) | 51 | 16.70 (12.20-36.40) |  | 417 | 15.90 (10.50-55.00) | 0.175 |
| **Child-Pugh score** | 50 | 8.00 (5.00-13.00) |  | 407 | 7.00 (5.00-13.00) | 0.085 |
| **Child-Pugh class A/B+C** | 50 | 11 (22.0%)/  39 (78.0%) |  | 407 | 145 (35.6%)/  262 (64.4%) | 0.055 |
| **MELD score** | 51 | 11.30 (6.43-38.01) |  | 409 | 10.37 (6.43-32.80) | 0.072 |
| **Source of variceal bleeding** |  |  |  |  |  |  |
| Esophageal varices (%) | 53 | 34 (64.2%) |  | 426 | 254 (59.6%) | 0.526 |
| Gastric varices (%) | 53 | 8 (15.1%) |  | 426 | 62 (14.6%) | 0.916 |
| Esophageal and gastric varices (%) | 53 | 11 (20.8%) |  | 426 | 110 (25.8%) | 0.423 |
| **Active variceal bleeding on endoscopy** | 55 | 6 (10.9%) |  | 430 | 27 (6.3%) | 0.199 |
| **Endoscopic variceal therapy** | 55 | 50 (90.9%) |  | 434 | 405 (93.3%) | 0.508 |
| **Surgery or interventional treatment** | 55 | 0 (0.0%) |  | 434 | 2 (0.5%) | 1.000 |
| **Abbreviations:** MELD, model for end-stage liver disease. | | | | | | |

| **Supplementary table 2. Propensity score matching analysis comparing the characteristics between patients undergoing endoscopy <12h and ≥12h according to the interval from the last presentation of AVB to endoscopy** | | | | |  |  |
| --- | --- | --- | --- | --- | --- | --- |
| **Variables** | **<12h group (n=46)** |  | **≥12h group (n=46)** | **P value** |  |  |
|  | **Median (Range) or Frequency (Percentage)** |  | **Median (Range) or Frequency (Percentage)** |  |  |  |
| **Age (years)** | 58.21 (27.49-88.73) |  | 55.29 (31.05-82.52) | 0.238 |  |  |
| **Male** | 31 (67.4%) |  | 39 (84.8%) | 0.051 |  |  |
| **Etiology of underlying liver diseases** |  |  |  |  |  |  |
| Hepatitis B virus | 13 (28.3%) |  | 20 (43.5%) | 0.128 |  |  |
| Hepatitis C virus | 9 (19.6%) |  | 2 (4.3%) | 0.050 |  |  |
| Alcohol abuse | 17 (37.0%) |  | 16 (34.8%) | 0.828 |  |  |
| **Hematemesis** | 26 (56.5%) |  | 28 (60.9%) | 0.672 |  |  |
| **Hepatocellular carcinoma** | 5 (10.9%) |  | 3 (6.5%) | 0.714 |  |  |
| **Hemodynamics** |  |  |  |  |  |  |
| Heart rate (beats per minute) | 80.00 (59.00-120.00) |  | 80.00 (66.00-126.00) | 0.975 |  |  |
| Heart rate >100 beats per minute | 5 (10.9%) |  | 5 (10.9%) | 1.000 |  |  |
| Systolic blood pressure (mmHg) | 117.00 (75.00-154.00) |  | 116.00 (75.00-169.00) | 0.991 |  |  |
| Systolic blood pressure <90mmHg | 3 (6.5%) |  | 3 (6.5%) | 1.000 |  |  |
| **Laboratory tests** |  |  |  |  |  |  |
| Hemoglobin (g/L) | 72.50 (47.00-157.00) |  | 78.00 (31.00-157.00) | 0.947 |  |  |
| White blood cell (10^12^/L) | 5.15 (1.30-46.10) |  | 4.75 (1.10-13.70) | 0.670 |  |  |
| Platelet count (10^9^/L) | 72.50 (22.00-457.00) |  | 72.00 (17.00-181.00) | 0.593 |  |  |
| Total bilirubin (μmol/L) | 20.45 (5.50-81.40) |  | 19.95 (6.90-85.30) | 0.673 |  |  |
| Albumin (g/L) | 30.05 (17.30-49.30) |  | 33.25 (10.00-48.40) | 0.199 |  |  |
| Alanine aminotransferase (U/L) | 25.00 (7.00-1064.00) |  | 26.00 (8.00-154.00) | 0.099 |  |  |
| Blood urea nitrogen (mmol/L) | 7.57 (3.04-28.25) |  | 7.99 (2.07-42.83) | 0.601 |  |  |
| Serum creatinine (μmol/L) | 58.00 (28.00-169.00) |  | 68.95 (40.00-715.00) | 0.070 |  |  |
| Sodium (mmol/L) | 139.10 (109.20-159.00) |  | 139.70 (130.70-150.00) | 0.560 |  |  |
| Prothrombin time (seconds) | 15.85 (12.20-36.40) |  | 15.30 (11.90-46.30) | 0.105 |  |  |
| **Child-Pugh score** | 7.00 (5.00-12.00) |  | 7.00 (5.00-13.00) | 0.112 |  |  |
| **Child-Pugh class A/B+C** | 11 (23.9%)/35 (76.1%) |  | 17 (37.0%)/29 (63.0%) | 0.174 |  |  |
| **MELD score** | 10.97 (6.43-30.12) |  | 10.24 (6.43-30.27) | 0.276 |  |  |
| **Source of variceal bleeding** |  |  |  |  |  |  |
| Esophageal varices (%) | 27 (61.4%) |  | 26 (61.9%) | 0.959 |  |  |
| Gastric varices (%) | 7 (15.9%) |  | 6 (14.3%) | 0.834 |  |  |
| Esophageal and gastric varices (%) | 9 (21.4%) |  | 10 (23.8%) | 0.905 |  |  |
| **Active variceal bleeding on endoscopy** | 4 (8.7%) |  | 6 (13.0%) | 0.739 |  |  |
| **Endoscopic variceal therapy** | 42 (91.3%) |  | 37 (80.4%) | 0.231 |  |  |
| **Surgery or interventional treatment** | 0 (0.0%) |  | 1 (2.2%) | 1.000 |  |  |
| **Abbreviations:** MELD, model for end-stage liver disease. | | | | |  |  |

| **Supplementary table 3. Difference between patients undergoing endoscopy <24h and ≥24h according to the interval from the last presentation of AVB to endoscopy** | | | | | | |  |
| --- | --- | --- | --- | --- | --- | --- | --- |
| **Variables** | **<24h group** | |  | **≥24h group** | | **P value** | |
|  | **No. Pts** | **Median (Range) or Frequency (Percentage)** |  | **No. Pts** | **Median (Range) or Frequency (Percentage)** |  |  |
| **Age (years)** | 114 | 59.70 (27.49-88.73) |  | 357 | 55.03 (6.28-92.31) | ***0.011*** | |
| **Male** | 114 | 77 (67.5%) |  | 357 | 255 (71.4%) | 0.429 | |
| **Etiology of underlying liver diseases** |  |  |  |  |  |  | |
| Hepatitis B virus | 114 | 39 (34.2%) |  | 357 | 154 (43.1%) | 0.092 | |
| Hepatitis C virus | 114 | 16 (14.0%) |  | 357 | 25 (7.0%) | ***0.020*** | |
| Alcohol abuse | 114 | 35 (30.70%) |  | 357 | 100 (28.0%) | 0.580 | |
| **Hematemesis** | 114 | 82 (71.9%) |  | 357 | 209 (58.5%) | ***0.010*** | |
| **Hepatocellular carcinoma** | 114 | 11 (9.6%) |  | 357 | 28 (7.8%) | 0.542 | |
| **Hemodynamics** |  |  |  |  |  |  | |
| Heart rate (beats per minute) | 114 | 80.00 (59.00-120.00) |  | 357 | 80.00 (44.00-148.00) | 0.831 | |
| Heart rate >100 beats per minute | 114 | 11 (9.6%) |  | 357 | 37 (10.4%) | 0.826 | |
| Systolic blood pressure (mmHg) | 114 | 114.50 (75.00-164.00) |  | 356 | 115.00 (75.00-176.00) | 0.163 | |
| Systolic blood pressure <90mmHg | 114 | 8 (7.0%) |  | 357 | 8 (2.2%) | ***0.015*** | |
| **Laboratory tests** |  |  |  |  |  |  | |
| Hemoglobin (g/L) | 114 | 72.00 (23.00-157.00) |  | 356 | 74.00 (30.00-158.00) | 0.389 | |
| White blood cell (10^12^/L) | 114 | 5.35 (1.30-46.10) |  | 356 | 4.20 (1.00-26.00) | ***0.002*** | |
| Platelet count (10^9^/L) | 114 | 71.00 (17.00-457.00) |  | 356 | 71.00 (15.00-435.00) | 0.871 | |
| Total bilirubin (μmol/L) | 113 | 21.70 (5.50-81.40) |  | 354 | 19.65 (3.30-107.00) | 0.265 | |
| Albumin (g/L) | 111 | 29.40 (10.00-49.30) |  | 350 | 30.90 (16.40-50.70) | 0.094 | |
| Alanine aminotransferase (U/L) | 113 | 22.00 (7.00-1064.00) |  | 352 | 22.08 (5.00-234.00) | 0.344 | |
| Blood urea nitrogen (mmol/L) | 111 | 8.52 (2.12-42.83) |  | 342 | 7.65 (2.03-28.90) | 0.082 | |
| Serum creatinine (μmol/L) | 111 | 62.00 (28.00-715.00) |  | 340 | 61.78 (25.00-501.52) | 0.375 | |
| Sodium (mmol/L) | 113 | 138.60 (109.20-159.00) |  | 349 | 138.5 (123.00-160.10) | 0.772 | |
| Prothrombin time (seconds) | 108 | 16.5 (12.20-36.40) |  | 344 | 15.90 (10.50-55.00) | 0.145 | |
| **Child-Pugh score** | 106 | 7.00 (5.00-13.00) |  | 335 | 7.00 (5.00-13.00) | ***0.017*** | |
| **Child-Pugh class A/B+C** | 106 | 26 (24.5%)/  80 (75.5%) |  | 335 | 123 (36.7%)/  212 (63.3%) | ***0.021*** | |
| **MELD score** | 108 | 10.88 (6.43-38.01) |  | 337 | 10.36 (6.43-32.80) | 0.061 | |
| **Source of variceal bleeding** |  |  |  |  |  |  | |
| Esophageal varices (%) | 109 | 68 (62.4%) |  | 350 | 211 (60.3%) | 0.695 | |
| Gastric varices (%) | 109 | 18 (16.5%) |  | 350 | 47 (13.4%) | 0.420 | |
| Esophageal and gastric varices (%) | 109 | 23 (21.1%) |  | 350 | 92 (26.3%) | 0.275 | |
| **Active variceal bleeding on endoscopy** | 113 | 16 (14.2%) |  | 354 | 15 (4.2%) | ***<0.001*** | |
| **Endoscopic variceal therapy** | 114 | 108 (94.7%) |  | 357 | 332 (93.0%) | 0.514 | |
| **Surgery or interventional treatment** | 114 | 0 (0.0%) |  | 357 | 1 (0.3%) | 1.000 | |
| Abbreviations: MELD, model for end-stage liver disease. | | | | | | |  |

| **Supplementary table 4. Propensity score matching analysis comparing the characteristics between patients undergoing endoscopy <24h and ≥24h according to the interval from the last presentation of AVB to endoscopy** | | | | |  |  |
| --- | --- | --- | --- | --- | --- | --- |
| **Variables** | **<24h group (n=97)** |  | **≥24h group (n=97)** | **P value** |  |  |
|  | **Median (Range) or Frequency (Percentage)** |  | **Median (Range) or Frequency (Percentage)** |  |  |  |
| **Age (years)** | 58.97 (27.49-88.73) |  | 57.57 (6.28-84.00) | 0.880 |  |  |
| **Male** | 63 (64.9%) |  | 63 (61.9%) | 0.655 |  |  |
| **Etiology of underlying liver diseases** |  |  |  |  |  |  |
| Hepatitis B virus | 32 (33.0%) |  | 45 (46.4%) | 0.056 |  |  |
| Hepatitis C virus | 14 (14.4%) |  | 6 (6.2%) | 0.059 |  |  |
| Alcohol abuse | 28 (28.9%) |  | 28 (28.9%) | 1.000 |  |  |
| **Hematemesis** | 67 (69.1%) |  | 65 (67.0%) | 0.758 |  |  |
| **Hepatocellular carcinoma** | 8 (8.2%) |  | 11 (11.3%) | 0.469 |  |  |
| **Hemodynamics** |  |  |  |  |  |  |
| Heart rate (beats per minute) | 80.00 (59.00-120.00) |  | 80.00 (56.00-130.00) | 0.586 |  |  |
| Heart rate >100 beats per minute | 9 (9.3%) |  | 8 (8.2%) | 0.800 |  |  |
| Systolic blood pressure (mmHg) | 116.00 (75.00-164.00) |  | 112.00 (75.00-169.00) | 0.589 |  |  |
| Systolic blood pressure <90mmHg | 5 (5.2%) |  | 4 (4.1%) | 1.000 |  |  |
| **Laboratory tests** |  |  |  |  |  |  |
| Hemoglobin (g/L) | 72.00 (23.00-157.00) |  | 74.00 (30.00-157.00) | 0.657 |  |  |
| White blood cell (10^12^/L) | 4.80 (1.30-46.10) |  | 4.30 (1.00-26.00) | 0.308 |  |  |
| Platelet count (10^9^/L) | 71.00 (17.00-457.00) |  | 73.00 (29.00-435.00) | 0.897 |  |  |
| Total bilirubin (μmol/L) | 20.60 (5.50-81.40) |  | 20.40 (5.20-90.40) | 0.748 |  |  |
| Albumin (g/L) | 29.80 (10.00-49.30) |  | 30.00 (16.40-48.40) | 0.438 |  |  |
| Alanine aminotransferase (U/L) | 22.00 (7.00-1064.00) |  | 25.00 (8.00-234.00) | 0.066 |  |  |
| Blood urea nitrogen (mmol/L) | 8.51 (2.12-42.83) |  | 7.87 (2.03-23.00) | 0.373 |  |  |
| Serum creatinine (μmol/L) | 61.50 (28.00-715.00) |  | 60.00 (25.00-234.00) | 0.522 |  |  |
| Sodium (mmol/L) | 138.60 (109.20-159.00) |  | 139.45 (127.80-150.00) | 0.457 |  |  |
| Prothrombin time (seconds) | 16.30 (12.20-36.40) |  | 16.30 (11.00-46.30) | 0.533 |  |  |
| **Child-Pugh score** | 7.00 (5.00-13.00) |  | 7.00 (5.00-13.00) | 0.353 |  |  |
| **Child-Pugh class A/B+C** | 24 (24.7%)/73 (75.3%) |  | 27 (27.8%)/70 (72.2%) | 0.625 |  |  |
| **MELD score** | 10.77 (6.43-30.27) |  | 10.74 (6.43-26.90) | 0.571 |  |  |
| **Source of variceal bleeding** |  |  |  |  |  |  |
| Esophageal varices (%) | 57 (61.3%) |  | 57 (61.3%) | 1.000 |  |  |
| Gastric varices (%) | 14 (15.1%) |  | 18 (19.4%) | 0.437 |  |  |
| Esophageal and gastric varices (%) | 22 (23.7%) |  | 18 (19.4%) | 0.475 |  |  |
| **Active variceal bleeding on endoscopy** | 10 (10.3%) |  | 12 (12.4%) | 0.651 |  |  |
| **Endoscopic variceal therapy** | 90 (92.8%) |  | 84 (86.6%) | 0.157 |  |  |
| **Surgery or interventional treatment** | 0 (0.0%) |  | 1 (1.0%) | 1.000 |  |  |
| **Abbreviations:** MELD, model for end-stage liver disease. | | | | |  |  |

| **Supplementary table 5. Difference between patients undergoing endoscopy <48h and ≥48h according to the interval from the last presentation of AVB to endoscopy** | | | | | | |
| --- | --- | --- | --- | --- | --- | --- |
| **Variables** | **<48h group** | |  | **≥48h group** | | **P value** |
|  | **No. Pts** | **Median (Range) or Frequency (Percentage)** |  | **No. Pts** | **Median (Range) or Frequency (Percentage)** |  |
| **Age (years)** | 226 | 58.06 (22.06-88.73) |  | 242 | 54.52 (6.28-92.31) | ***0.038*** |
| **Male** | 226 | 76 (33.6%) |  | 242 | 71 (29.3%) | 0.318 |
| **Etiology of underlying liver diseases** |  |  |  |  |  |  |
| Hepatitis B virus | 226 | 89 (39.4%) |  | 242 | 102 (42.1%) | 0.543 |
| Hepatitis C virus | 226 | 28 (12.4%) |  | 242 | 16 (6.6%) | ***0.032*** |
| Alcohol abuse | 226 | 62 (27.4%) |  | 242 | 72 (29.8%) | 0.579 |
| **Hematemesis** | 226 | 159 (70.4%) |  | 242 | 141 (58.3%) | ***0.006*** |
| **Hepatocellular carcinoma** | 226 | 21 (9.3%) |  | 242 | 20 (8.3%) | 0.694 |
| **Hemodynamics** |  |  |  |  |  |  |
| Heart rate (beats per minute) | 226 | 80.00 (59.00-130.00) |  | 242 | 80.00 (44.00-148.00) | 0.602 |
| Heart rate >100 beats per minute | 226 | 20 (8.8%) |  | 242 | 25 (10.3%) | 0.587 |
| Systolic blood pressure (mmHg) | 226 | 115.00 (75.00-166.00) |  | 241 | 116.00 (75.00-176.00) | 0.069 |
| Systolic blood pressure <90mmHg | 226 | 11 (4.9%) |  | 241 | 3 (1.2%) | ***0.028*** |
| **Laboratory tests** |  |  |  |  |  |  |
| Hemoglobin (g/L) | 226 | 72.50 (23.00-157.00) |  | 241 | 74.00 (31.00-137.00) | 0.702 |
| White blood cell (10^12^/L) | 226 | 5.10 (1.00-46.10) |  | 241 | 4.20 (1.10-26.00) | ***0.003*** |
| Platelet count (10^9^/L) | 226 | 73.00 (17.00-457.00) |  | 241 | 72.00 (15.00-435.00) | 0.710 |
| Total bilirubin (μmol/L) | 224 | 20.05 (3.90-187.40) |  | 239 | 19.80 (3.30-107.00) | 0.928 |
| Albumin (g/L) | 219 | 30.00 (10.00-49.30) |  | 240 | 30.90 (16.40-50.70) | 0.173 |
| Alanine aminotransferase (U/L) | 223 | 22.00 (5.00-1064.00) |  | 238 | 22.32 (6.00-152.11) | 0.387 |
| Blood urea nitrogen (mmol/L) | 217 | 8.50 (1.54-42.83) |  | 230 | 7.51 (2.03-28.90) | ***0.020*** |
| Serum creatinine (μmol/L) | 217 | 62.50 (28.00-715.00) |  | 228 | 59.26 (25.00-220.30) | 0.084 |
| Sodium (mmol/L) | 223 | 138.5 (109.20-159.00) |  | 235 | 138.60 (127.80-160.10) | 0.638 |
| Prothrombin time (seconds) | 215 | 16.00 (12.20-36.40) |  | 232 | 15.90 (10.50-55.00) | 0.369 |
| **Child-Pugh score** | 207 | 7.00 (5.00-13.00) |  | 228 | 7.00 (5.00-13.00) | ***0.028*** |
| **Child-Pugh class A/B+C** | 207 | 56 (27.1%)/151 (72.9%) |  | 228 | 85 (37.3%)/143 (62.7%) | ***0.023*** |
| **MELD score** | 212 | 10.71 (6.43-38.01) |  | 226 | 10.44 (6.43-32.80) | 0.178 |
| **Source of variceal bleeding** |  |  |  |  |  |  |
| Esophageal varices (%) | 217 | 131 (60.4%) |  | 240 | 146 (60.8%) | 0.919 |
| Gastric varices (%) | 217 | 36 (16.6%) |  | 240 | 32 (13.3%) | 0.329 |
| Esophageal and gastric varices (%) | 217 | 50 (23.0%) |  | 240 | 62 (25.8%) | 0.488 |
| **Active variceal bleeding on endoscopy** | 224 | 29 (12.9%) |  | 241 | 7 (2.9%) | ***<0.001*** |
| **Endoscopic variceal therapy** | 226 | 211 (93.4%) |  | 242 | 225 (93.0%) | 0.868 |
| **Surgery or interventional treatment** | 226 | 2 (0.9%) |  | 242 | 0 (0.0%) | 0.233 |
| **Abbreviations:** MELD, model for end-stage liver disease. | | | | | | |

| **Supplementary table 6. Propensity score matching analysis comparing the characteristics between patients undergoing endoscopy <48h and ≥48h according to the interval from the last presentation of AVB to endoscopy** | | | | |
| --- | --- | --- | --- | --- |
| **Variables** | **<48h group (n=165)** |  | **≥48h group (n=165)** | **P value** |
|  | **Median (Range) or Frequency (Percentage)** |  | **Median (Range) or Frequency (Percentage)** |  |
| **Age (years)** | 57.54 (22.06-88.73) |  | 54.60 (6.28-92.31) | 0.261 |
| **Male** | 109 (66.1%) |  | 110 (66.7%) | 0.907 |
| **Etiology of underlying liver diseases** |  |  |  |  |
| Hepatitis B virus | 63 (38.2%) |  | 61 (37.0%) | 0.820 |
| Hepatitis C virus | 22 (13.3%) |  | 13 (7.9%) | 0.108 |
| Alcohol abuse | 44 (26.7%) |  | 53 (32.1%) | 0.277 |
| **Hematemesis** | 113 (68.5%) |  | 110 (66.7%) | 0.724 |
| **Hepatocellular carcinoma** | 15 (9.1%) |  | 16 (9.7%) | 0.850 |
| **Hemodynamics** |  |  |  |  |
| Heart rate (beats per minute) | 80.00 (59.00-130.00) |  | 80.00 (44.00-124.00) | 0.698 |
| Heart rate >100 beats per minute | 17 (10.3%) |  | 16 (9.7%) | 0.854 |
| Systolic blood pressure (mmHg) | 116.00 (85.00-166.00) |  | 114.00 (75.00-176.00) | 0.964 |
| Systolic blood pressure <90mmHg | 1 (0.6%) |  | 2 (1.2%) | 1.000 |
| **Laboratory tests** |  |  |  |  |
| Hemoglobin (g/L) | 73.00 (23.00-157.00) |  | 73.00 (31.00-133.00) | 0.764 |
| White blood cell (10^12^/L) | 4.60 (1.00-26.30) |  | 4.30 (1.10-26.00) | 0.435 |
| Platelet count (10^9^/L) | 73.00 (17.00-457.00) |  | 72.00 (29.00-435.00) | 0.714 |
| Total bilirubin (μmol/L) | 19.7 (5.30-187.4) |  | 20.70 (3.30-107.00) | 0.280 |
| Albumin (g/L) | 29.70 (10.00-49.30) |  | 29.30 (16.40-47.00) | 0.812 |
| Alanine aminotransferase (U/L) | 23.00 (5.00-617.99) |  | 23.00 (6.79-152.11) | 0.211 |
| Blood urea nitrogen (mmol/L) | 8.14 (1.54-42.83) |  | 7.98 (2.03-28.90) | 0.294 |
| Serum creatinine (μmol/L) | 62.00 (28.00-715.00) |  | 58.00 (25.00-220.30) | 0.111 |
| Sodium (mmol/L) | 138.50 (109.20-159.00) |  | 138.50 (127.80-160.10) | 0.601 |
| Prothrombin time (seconds) | 16.4 (12.20-36.10) |  | 16.10 (10.80-55.00) | 0.388 |
| **Child-Pugh score** | 7.00 (5.00-13.00) |  | 7.00 (5.00-13.00) | 0.899 |
| **Child-Pugh class A/B+C** | 46 (27.9%)/119 (72.1%) |  | 51 (30.9%)/114 (69.1%) | 0.546 |
| **MELD score** | 10.68 (6.43-30.27) |  | 10.82 (6.43-32.80) | 0.740 |
| **Source of variceal bleeding** |  |  |  |  |
| Esophageal varices (%) | 97 (61.0%) |  | 97 (61.0%) | 0.995 |
| Gastric varices (%) | 23 (14.5%) |  | 24 (14.6%) | 0.966 |
| Esophageal and gastric varices (%) | 39 (24.5%) |  | 40 (24.4%) | 0.977 |
| **Active variceal bleeding on endoscopy** | 8 (4.8%) |  | 7 (4.2%) | 0.792 |
| **Endoscopic variceal therapy** | 155 (93.9%) |  | 153 (92.7%) | 0.659 |
| **Surgery or interventional treatment** | 1 (0.6%) |  | 0 (0.0%) | 1.000 |
| **Abbreviations:** MELD, model for end-stage liver disease. | | | | |

| **Supplementary table 7. Difference between patients undergoing endoscopy <12h and ≥12h according to the interval from the admission to endoscopy** | | | | | | |  |
| --- | --- | --- | --- | --- | --- | --- | --- |
| **Variables** | **<12h group** | |  | **≥12h group** | | **P value** | |
|  | **No. Pts** | **Median (Range) or Frequency (Percentage)** |  | **No. Pts** | **Median (Range) or Frequency (Percentage)** |  |  |
| **Age (years)** | 75 | 59.76 (27.49-82.60) |  | 453 | 55.14 (6.28-92.31) | ***0.020*** | |
| **Male** | 75 | 48 (64.0%) |  | 453 | 323 (71.3%) | 0.200 | |
| **Etiology of underlying liver diseases** |  |  |  |  |  |  | |
| Hepatitis B virus | 75 | 26 (34.7%) |  | 453 | 192 (42.4%) | 0.209 | |
| Hepatitis C virus | 75 | 13 (17.3%) |  | 453 | 34 (7.5%) | ***0.006*** | |
| Alcohol abuse | 75 | 22 (29.3%) |  | 453 | 127 (28.0%) | 0.817 | |
| **Hematemesis** | 75 | 53 (70.7%) |  | 453 | 276 (60.9%) | 0.107 | |
| **Hepatocellular carcinoma** | 75 | 10 (13.3%) |  | 453 | 37 (8.2%) | 0.146 | |
| **Hemodynamics** |  |  |  |  |  |  | |
| Heart rate (beats per minute) | 75 | 82.00 (60.00-120.00) |  | 453 | 80.00 (44.00-148.00) | 0.276 | |
| Heart rate >100 beats per minute | 75 | 6 (8.0%) |  | 452 | 45 (9.9%) | 0.599 | |
| Systolic blood pressure (mmHg) | 75 | 116.00 (75.00-164.00) |  | 452 | 115.00 (75.00-176.00) | 0.522 | |
| Systolic blood pressure <90mmHg | 75 | 5 (6.7%) |  | 452 | 13 (2.9%) | 0.094 | |
| **Laboratory tests** |  |  |  |  |  |  | |
| Hemoglobin (g/L) | 75 | 73.00 (23.00-157.00) |  | 452 | 74.00 (30.00-158.00) | 0.829 | |
| White blood cell (10^12^/L) | 75 | 5.30 (1.70-46.10) |  | 452 | 4.30 (1.00-26.00) | ***0.017*** | |
| Platelet count (10^9^/L) | 75 | 62.00 (22.00-165.00) |  | 452 | 73.00 (15.00-457.00) | 0.172 | |
| Total bilirubin (μmol/L) | 75 | 23.70 (3.90-187.40) |  | 448 | 19.75 (3.30-107.00) | ***0.047*** | |
| Albumin (g/L) | 73 | 29.30 (10.50-49.30) |  | 445 | 30.70 (10.00-50.70) | 0.081 | |
| Alanine aminotransferase (U/L) | 75 | 25.00 (8.00-1064.00) |  | 446 | 22.00 (5.00-617.99) | 0.414 | |
| Blood urea nitrogen (mmol/L) | 71 | 8.51 (2.43-42.83) |  | 435 | 7.80 (1.54-28.90) | ***0.045*** | |
| Serum creatinine (μmol/L) | 71 | 62.50 (28.00-715.00) |  | 433 | 61.96 (25.00-501.52) | 0.557 | |
| Sodium (mmol/L) | 75 | 138.90 (109.20-145.40) |  | 443 | 138.50 (123.00-160.10) | 0.390 | |
| Prothrombin time (seconds) | 66 | 16.7 (12.20-36.40) |  | 438 | 15.90 (10.50-55.00) | 0.072 | |
| **Child-Pugh score** | 65 | 8.00 (5.00-13.00) |  | 427 | 7.00 (5.00-13.00) | ***0.001*** | |
| **Child-Pugh class A/B+C** | 65 | 11 (16.9%)/  54 (83.1%) |  | 427 | 151 (35.4%)/  276 (64.6%) | ***0.003*** | |
| **MELD score** | 66 | 12.76 (6.43-38.01) |  | 430 | 10.42 (6.43-32.80) | ***0.002*** | |
| **Source of variceal bleeding** |  |  |  |  |  |  | |
| Esophageal varices (%) | 72 | 43 (59.7%) |  | 441 | 264 (59.9%) | 0.982 | |
| Gastric varices (%) | 72 | 12 (16.7%) |  | 441 | 65 (14.7%) | 0.671 | |
| Esophageal and gastric varices (%) | 72 | 17 (23.6%) |  | 441 | 112 (25.4%) | 0.746 | |
| **Active variceal bleeding on endoscopy** | 74 | 10 (13.5%) |  | 448 | 28 (6.3%) | ***0.026*** | |
| **Endoscopic variceal therapy** | 75 | 70 (93.3%) |  | 453 | 423 (93.4%) | 0.989 | |
| **Surgery or interventional treatment** | 75 | 1 (1.3%) |  | 453 | 2 (0.4%) | 0.369 | |
| **Abbreviations:** MELD, model for end-stage liver disease. | | | | | | |  |

| **Supplementary table 8. Propensity score matching analysis comparing the characteristics between patients undergoing endoscopy <12h and ≥12h according to the interval from the admission to endoscopy** | | | | |
| --- | --- | --- | --- | --- |
| **Variables** | **<12h group (n=63)** |  | **≥12h group (n=63)** | **P**  **value** |
|  | **Median (Range) or Frequency (Percentage)** |  | **Median (Range) or Frequency (Percentage)** |  |
| **Age (years)** | 58.03 (27.49-78.37) |  | 57.65 (38.52-84.00) | 0.903 |
| **Male** | 41 (65.1%) |  | 41 (65.1%) | 1.000 |
| **Etiology of underlying liver diseases** |  |  |  |  |
| Hepatitis B virus | 21 (33.3%) |  | 21 (33.3%) | 1.000 |
| Hepatitis C virus | 10 (15.9%) |  | 3 (4.8%) | 0.076 |
| Alcohol abuse | 18 (28.6%) |  | 17 (27.0%) | 0.842 |
| **Hematemesis** | 43 (68.3%) |  | 44 (69.8%) | 0.847 |
| **Hepatocellular carcinoma** | 7 (11.1%) |  | 6 (9.5%) | 0.770 |
| **Hemodynamics** |  |  |  |  |
| Heart rate (beats per minute) | 82.00 (60.00-120.00) |  | 80.00 (66.00-126.00) | 0.263 |
| Heart rate >100 beats per minute | 5 (7.9%) |  | 4 (6.3%) | 1.000 |
| Systolic blood pressure (mmHg) | 118.00 (75.00-164.00) |  | 120.00 (83.00-169.00) | 0.938 |
| Systolic blood pressure <90mmHg | 3 (4.8%) |  | 4 (6.3%) | 1.000 |
| **Laboratory tests** |  |  |  |  |
| Hemoglobin (g/L) | 72.00 (23.00-157.00) |  | 73.00 (31.00-157.00) | 0.617 |
| White blood cell (10^12^/L) | 5.30 (1.70-46.10) |  | 4.60 (1.10-17.50) | 0.593 |
| Platelet count (10^9^/L) | 61.00 (22.00-165.00) |  | 69.00 (17.00-289.00) | 0.714 |
| Total bilirubin (μmol/L) | 24.40 (5.50-187.40) |  | 24.10 (6.90-107.00) | 0.323 |
| Albumin (g/L) | 28.70 (10.50-49.30) |  | 29.70 (10.00-48.40) | 0.725 |
| Alanine aminotransferase (U/L) | 25.00 (8.00-1064.00) |  | 24.00 (7.00-234.00) | 0.771 |
| Blood urea nitrogen (mmol/L) | 8.51 (2.43-42.83) |  | 8.06 (2.07-17.64) | 0.223 |
| Serum creatinine (μmol/L) | 61.00 (28.00-715.00) |  | 61.00 (31.00-222.00) | 0.586 |
| Sodium (mmol/L) | 139.10 (109.20-145.40) |  | 139.30 (128.00-149.00) | 0.967 |
| Prothrombin time (seconds) | 16.70 (12.20-36.40) |  | 16.60 (11.90-33.70) | 0.610 |
| **Child-Pugh score** | 8.00 (5.00-12.00) |  | 8.00 (5.00-13.00) | 0.772 |
| **Child-Pugh class A/B+C** | 11 (17.5%)/52 (82.5%) |  | 13 (20.6%)/50 (79.4%) | 0.821 |
| **MELD score** | 12.66 (6.43-30.27) |  | 11.80 (6.43-23.89) | 0.586 |
| **Source of variceal bleeding** |  |  |  |  |
| Esophageal varices (%) | 34 (55.7%) |  | 29 (50.0%) | 0.531 |
| Gastric varices (%) | 11 (18.0%) |  | 11 (19.0%) | 0.896 |
| Esophageal and gastric varices (%) | 16 (26.2%) |  | 18 (31.0%) | 0.562 |
| **Active variceal bleeding on endoscopy** | 9 (14.3%) |  | 10 (15.9%) | 0.803 |
| **Endoscopic variceal therapy** | 57 (90.5%) |  | 52 (82.5%) | 0.192 |
| **Surgery or interventional treatment** | 1 (1.6%) |  | 1 (1.6%) | 1.000 |
| **Abbreviations:** MELD, model for end-stage liver disease. | | | | |

| **Supplementary table 9. Difference between patients undergoing endoscopy <24h and ≥24h according to the interval from the admission to endoscopy** | | | | | | |
| --- | --- | --- | --- | --- | --- | --- |
| **Variables** | **<24h group** | |  | **≥24h group** | | **P value** |
|  | **No. Pts** | **Median (Range) or Frequency (Percentage)** |  | **No. Pts** | **Median (Range) or Frequency (Percentage)** |  |
| **Age (years)** | 175 | 57.54 (22.06-86.00) |  | 349 | 54.73 (6.28-92.31) | ***0.030*** |
| **Male** | 175 | 124 (70.9%) |  | 349 | 245 (70.2%) | 0.877 |
| **Etiology of underlying liver diseases** |  |  |  |  |  |  |
| Hepatitis B virus | 175 | 69 (39.4%) |  | 349 | 147 (42.1%) | 0.555 |
| Hepatitis C virus | 175 | 19 (10.90%) |  | 349 | 28 (8.0%) | 0.284 |
| Alcohol abuse | 175 | 52 (29.7%) |  | 349 | 95 (27.2%) | 0.549 |
| **Hematemesis** | 175 | 112 (64.0%) |  | 349 | 216 (61.9%) | 0.638 |
| **Hepatocellular carcinoma** | 175 | 17 (9.7%) |  | 349 | 31 (8.9%) | 0.756 |
| **Hemodynamics** |  |  |  |  |  |  |
| Heart rate (beats per minute) | 175 | 80.00 (58.00-120.00) |  | 349 | 80.00 (44.00-148.00) | 0.404 |
| Heart rate >100 beats per minute | 175 | 14 (8.0%) |  | 349 | 37 (10.6%) | 0.343 |
| Systolic blood pressure (mmHg) | 175 | 116.00 (75.00-176.00) |  | 348 | 114.50 (75.00-169.00) | 0.967 |
| Systolic blood pressure <90mmHg | 175 | 9 (5.1%) |  | 348 | 8 (2.3%) | 0.084 |
| **Laboratory tests** |  |  |  |  |  |  |
| Hemoglobin (g/L) | 175 | 73.00 (23.00-157.00) |  | 348 | 74.00 (30.00-158.00) | 0.725 |
| White blood cell (10^12^/L) | 175 | 4.60 (1.10-46.10) |  | 348 | 4.30 (1.00-26.00) | 0.153 |
| Platelet count (10^9^/L) | 175 | 72.00 (18.00-446.00) |  | 348 | 71.50 (15.00-457.00) | 0.833 |
| Total bilirubin (μmol/L) | 175 | 19.70 (3.90-187.40) |  | 345 | 19.90 (3.30-107.00) | 0.971 |
| Albumin (g/L) | 171 | 29.70 (10.50-49.30) |  | 343 | 30.90 (10.00-50.70) | 0.115 |
| Alanine aminotransferase (U/L) | 175 | 23.00 (6.00-1064.00) |  | 343 | 22.00 (5.00-154.00) | 0.889 |
| Blood urea nitrogen (mmol/L) | 170 | 8.42 (2.12-42.83) |  | 333 | 7.87 (1.54-28.90) | 0.186 |
| Serum creatinine (μmol/L) | 170 | 64.50 (28.00-715.00) |  | 331 | 61.10 (25.00-501.52) | 0.065 |
| Sodium (mmol/L) | 175 | 138.30 (109.20-159.00) |  | 339 | 138.60 (127.80-160.10) | 0.482 |
| Prothrombin time (seconds) | 164 | 16.25 (12.20-36.40) |  | 336 | 15.90 (10.50-55.00) | 0.356 |
| **Child-Pugh score** | 162 | 8.00 (5.00-13.00) |  | 327 | 7.00 (5.00-13.00) | ***0.020*** |
| **Child-Pugh class A/B+C** | 162 | 42 (25.9%)/  120 (74.1%) |  | 327 | 117 (35.8%)/  210 (64.2%) | ***0.029*** |
| **MELD score** | 165 | 10.68 (6.43-38.01) |  | 328 | 10.48 (6.43-32.80) | 0.269 |
| **Source of variceal bleeding** |  |  |  |  |  |  |
| Esophageal varices (%) | 172 | 109 (63.4%) |  | 338 | 199 (58.9%) | 0.326 |
| Gastric varices (%) | 172 | 26 (15.1%) |  | 338 | 50 (14.8%) | 0.923 |
| Esophageal and gastric varices (%) | 172 | 37 (21.5%) |  | 338 | 89 (26.3%) | 0.233 |
| **Active variceal bleeding on endoscopy** | 174 | 15 (8.6%) |  | 344 | 23 (6.7%) | 0.425 |
| **Endoscopic variceal therapy** | 175 | 164 (93.7%) |  | 349 | 326 (93.4%) | 0.894 |
| **Surgery or interventional treatment** | 175 | 1 (0.6%) |  | 349 | 2 (0.6%) | 1.000 |
| **Abbreviations:** MELD, model for end-stage liver disease. | | | | | | |

| **Supplementary table 10. Propensity score matching analysis comparing the characteristics between patients undergoing endoscopy <24h and ≥24h according to the interval from the admission to endoscopy** | | | | |
| --- | --- | --- | --- | --- |
| **Variables** | **<24h group (n=155)** |  | **≥24h group (n=155)** | **P value** |
|  | **Median (Range) or Frequency (Percentage)** |  | **Median (Range) or Frequency (Percentage)** |  |
| **Age (years)** | 56.98 (22.06-86.00） |  | 55.68 (21.95-82.52) | 0.929 |
| **Male** | 110 (71.0%) |  | 105 (67.7%) | 0.538 |
| **Etiology of underlying liver diseases** |  |  |  |  |
| Hepatitis B virus | 60 (38.7%) |  | 67 (43.2%) | 0.419 |
| Hepatitis C virus | 16 (10.3%) |  | 13 (8.4%) | 0.558 |
| Alcohol abuse | 42 (27.1%) |  | 55 (35.5%) | 0.111 |
| **Hematemesis** | 98 (63.2%) |  | 97 (62.6%) | 1.000 |
| **Hepatocellular carcinoma** | 14 (9.0%) |  | 20 (12.9%) | 0.275 |
| **Hemodynamics** |  |  |  |  |
| Heart rate (beats per minute) | 80.00 (58.00-120.00) |  | 80.00 (54.00-130.00) | 0.793 |
| Heart rate >100 beats per minute | 12 (7.7%) |  | 14 (9.0%) | 0.682 |
| Systolic blood pressure (mmHg) | 118.00 (75.00-176.00) |  | 114.00 (77.00-169.00) | 0.418 |
| Systolic blood pressure <90mmHg | 3 (1.9%) |  | 3 (1.9%) | 1.000 |
| **Laboratory tests** |  |  |  |  |
| Hemoglobin (g/L) | 75.00 (23.00-157.00) |  | 70.00 (30.00-157.00) | 0.072 |
| White blood cell (10^12^/L) | 4.50 (1.10-46.10) |  | 4.30 (1.00-26.00) | 0.293 |
| Platelet count (10^9^/L) | 73.00 (18.00-446.00) |  | 71.00 (17.00-374.00) | 0.909 |
| Total bilirubin (μmol/L) | 18.80 (5.50-187.40) |  | 20.10 (3.30-107.00) | 0.533 |
| Albumin (g/L) | 29.80 (10.50-49.30) |  | 30.10 (10.00-48.40) | 0.564 |
| Alanine aminotransferase (U/L) | 23.00 (6.00-1064.00) |  | 23.00 (7.00-154.00) | 0.818 |
| Blood urea nitrogen (mmol/L) | 8.26 (2.12-42.83) |  | 7.66 (1.54-28.90) | 0.178 |
| Serum creatinine (μmol/L) | 64.00 (28.00-715.00) |  | 60.00 (25.00-234.00) | 0.054 |
| Sodium (mmol/L) | 138.50 (109.20-159.00) |  | 139.25 (127.80-160.10) | 0.341 |
| Prothrombin time (seconds) | 16.20 (12.20-36.40) |  | 15.80 (10.80-46.30) | 0.370 |
| **Child-Pugh score** | 8.00 (5.00-12.00) |  | 7.00 (5.00-13.00) | 0.929 |
| **Child-Pugh class A/B+C** | 42 (27.1%)/113 (72.9%) |  | 44 (28.4%)/111 (71.6%) | 0.800 |
| **MELD score** | 10.59 (6.43-30.27) |  | 10.74 (6.43-26.90) | 0.513 |
| **Source of variceal bleeding** |  |  |  |  |
| Esophageal varices (%) | 96 (62.7%) |  | 79 (53.4%) | 0.100 |
| Gastric varices (%) | 23 (15.0%) |  | 24 (16.2%) | 0.777 |
| Esophageal and gastric varices (%) | 34 (22.2%) |  | 45 (30.4%) | 0.107 |
| **Active variceal bleeding on endoscopy** | 14 (9.0%) |  | 15 (9.7%) | 0.845 |
| **Endoscopic variceal therapy** | 146 (94.2%) |  | 139 (89.7%) | 0.144 |
| **Surgery or interventional treatment** | 1 (0.6%) |  | 2 (1.3%) | 1.000 |
| **Abbreviations:** MELD, model for end-stage liver disease. | | | | |

| **Supplementary table 11. Difference between patients undergoing endoscopy <48h and ≥48h according to the interval from the admission to endoscopy** | | | | | | |  |
| --- | --- | --- | --- | --- | --- | --- | --- |
| **Variables** | **<48h group** | |  | **≥48h group** | | **P value** | |
|  | **No. Pts** | **Median (Range) or Frequency (Percentage)** |  | **No. Pts** | **Median (Range) or Frequency (Percentage)** |  |  |
| **Age (years)** | 289 | 57.21 (21.95-88.73) |  | 236 | 54.37 (6.28-92.31) | ***0.030*** | |
| **Male** | 289 | 205 (70.9%) |  | 236 | 166 (70.3%) | 0.882 | |
| **Etiology of underlying liver diseases** |  |  |  |  |  |  | |
| Hepatitis B virus | 289 | 118 (40.8%) |  | 236 | 100 (42.4%) | 0.721 | |
| Hepatitis C virus | 289 | 31 (10.7%) |  | 236 | 15 (6.4%) | 0.078 | |
| Alcohol abuse | 289 | 76 (26.3%) |  | 236 | 71 (30.1%) | 0.336 | |
| **Hematemesis** | 289 | 182 (63.0%) |  | 236 | 144 (61.0%) | 0.645 | |
| **Hepatocellular carcinoma** | 289 | 27 (9.3%) |  | 236 | 19 (8.1%) | 0.603 | |
| **Hemodynamics** |  |  |  |  |  |  | |
| Heart rate (beats per minute) | 289 | 80.00 (58.00-130.00) |  | 236 | 80.00 (44.00-148.00) | 0.084 | |
| Heart rate >100 beats per minute | 289 | 22 (7.6%) |  | 236 | 28 (11.9%) | 0.099 | |
| Systolic blood pressure (mmHg) | 288 | 115.50 (75.00-176.00) |  | 236 | 115.00 (75.00-165.00) | 0.558 | |
| Systolic blood pressure <90mmHg | 288 | 12 (4.2%) |  | 236 | 6 (2.5%) | 0.310 | |
| **Laboratory tests** |  |  |  |  |  |  | |
| Hemoglobin (g/L) | 289 | 74.00 (23.00-158.00) |  | 235 | 73.00 (31.00-141.00) | 0.123 | |
| White blood cell (10^12^/L) | 289 | 4.50 (1.10-46.10) |  | 235 | 4.40 (1.00-26.00) | 0.937 | |
| Platelet count (10^9^/L) | 289 | 71.00 (18.00-457.00) |  | 235 | 75.00 (15.00-435.00) | 0.237 | |
| Total bilirubin (μmol/L) | 286 | 20.30 (3.90-187.40) |  | 234 | 19.70 (3.30-107.00) | 0.619 | |
| Albumin (g/L) | 282 | 30.45 (10.50-50.70) |  | 233 | 30.70 (10.00-45.60) | 0.613 | |
| Alanine aminotransferase (U/L) | 284 | 22.50 (5.00-1064.00) |  | 234 | 22.57 (6.00-154.00) | 0.971 | |
| Blood urea nitrogen (mmol/L) | 280 | 8.03 (2.12-42.83) |  | 223 | 8.11 (1.54-28.90) | 0.822 | |
| Serum creatinine (μmol/L) | 278 | 62.06 (28.00-715.00) |  | 223 | 60.00 (25.00-220.30) | 0.151 | |
| Sodium (mmol/L) | 284 | 138.55 (109.20-159.00) |  | 231 | 138.50 (127.80-160.10) | 0.986 | |
| Prothrombin time (seconds) | 276 | 16.00 (10.50-36.40) |  | 225 | 16.10 (10.80-55.00) | 0.477 | |
| **Child-Pugh score** | 268 | 7.00 (5.00-13.00) |  | 221 | 7.00 (5.00-13.00) | 0.542 | |
| **Child-Pugh class A/B+C** | 268 | 83 (31.0%)/79 (35.7%) |  | 221 | 79 (35.7%)/142 (64.3%) | 0.264 | |
| **MELD score** | 272 | 10.45 (6.43-38.01) |  | 221 | 10.58 (6.43-32.80) | 0.978 | |
| **Source of variceal bleeding** |  |  |  |  |  |  | |
| Esophageal varices (%) | 280 | 178 (63.6%) |  | 231 | 130 (56.3%) | 0.094 | |
| Gastric varices (%) | 280 | 40 (14.3%) |  | 231 | 36 (15.6%) | 0.681 | |
| Esophageal and gastric varices (%) | 280 | 62 (22.1%) |  | 231 | 65 (28.1%) | 0.119 | |
| **Active variceal bleeding on endoscopy** | 287 | 26 (9.1%) |  | 232 | 11 (4.7%) | 0.057 | |
| **Endoscopic variceal therapy** | 289 | 269 (93.1%) |  | 236 | 222 (94.1%) | 0.647 | |
| **Surgery or interventional treatment** | 289 | 2 (0.7%) |  | 236 | 1 (0.4%) | 1.000 | |
| **Abbreviations:** MELD, model for end-stage liver disease. | | | | | | |  |

| **Supplementary table 12. Propensity score matching analysis comparing the characteristics between patients undergoing endoscopy <48h and ≥48h according to the interval from the admission to endoscopy** | | | | |
| --- | --- | --- | --- | --- |
| **Variables** | **<48h group (n=199)** |  | **≥48h group (n=199)** | **P value** |
|  | **Median (Range) or Frequency (Percentage)** |  | **Median (Range) or Frequency (Percentage)** |  |
| **Age (years)** | 56.73 (21.95-84.00) |  | 54.51 (6.28-92.31) | 0.406 |
| **Male** | 143 (71.9%) |  | 140 (70.4%) | 0.740 |
| **Etiology of underlying liver diseases** |  |  |  |  |
| Hepatitis B virus | 80 (40.2%) |  | 82 (41.2%) | 0.838 |
| Hepatitis C virus | 21 (10.6%) |  | 14 (7.0%) | 0.215 |
| Alcohol abuse | 55 (27.6%) |  | 60 (30.2%) | 0.580 |
| **Hematemesis** | 122 (61.3%) |  | 123 (61.8%) | 0.918 |
| **Hepatocellular carcinaoma** | 19 (9.5%) |  | 14 (7.0%) | 0.363 |
| **Hemodynamics** |  |  |  | 0.629 |
| Heart rate (beats per minute) | 80.00 (58.00-120.00) |  | 80.00 (44.00-126.00) | 0.757 |
| Heart rate >100 beats per minute | 18 (9.0%) |  | 15 (7.5%) | 0.586 |
| Systolic blood pressure (mmHg) | 116.00 (83.00-176.00) |  | 115.00 (75.00-165.00) | 0.675 |
| Systolic blood pressure <90mmHg | 1 (0.5%) |  | 4 (2.0%) | 0.372 |
| **Laboratory tests** |  |  |  |  |
| Hemoglobin (g/L) | 75.00 (31.00-158.00) |  | 73.00 (31.00-131.00) | 0.082 |
| White blood cell (10^12^/L) | 4.50 (1.10-46.10) |  | 4.60 (1.00-26.00) | 0.671 |
| Platelet count (10^9^/L) | 71.00 (18.00-446.00) |  | 76.00 (15.00-435.00) | 0.191 |
| Total bilirubin (μmol/L) | 20.30 (5.30-187.40) |  | 19.80 (3.30-107.00) | 0.725 |
| Albumin (g/L) | 29.90 (10.50-50.70) |  | 30.60 (16.40-45.60) | 0.884 |
| Alanine aminotransferase (U/L) | 22.81 (5.00-1064.00) |  | 22.73 (6.79-152.11) | 0.753 |
| Blood urea nitrogen (mmol/L) | 7.71 (2.12-22.08) |  | 7.98 (1.54-28.90) | 0.256 |
| Serum creatinine (μmol/L) | 61.60 (28.00-179.50) |  | 60.00 (25.00-220.30) | 0.445 |
| Sodium (mmol/L) | 138.50 (109.20-159.00) |  | 138.60 (127.80-160.10) | 0.602 |
| Prothrombin time (seconds) | 16.00 (10.50-36.40) |  | 16.20 (10.80-33.70) | 0.577 |
| **Child-Pugh score** | 7.00 (5.00-12.00) |  | 7.00 (5.00-13.00) | 0.551 |
| **Child-Pugh class A/B+C** | 63 (31.7%)/136 (68.3%) |  | 72 (36.2%)/127 (63.8%) | 0.341 |
| **MELD score** | 10.37 (6.43-30.12) |  | 10.58 (6.43-25.64) | 0.760 |
| **Source of variceal bleeding** |  |  |  |  |
| Esophageal varices (%) | 124 (63.6%) |  | 109 (55.6%) | 0.108 |
| Gastric varices (%) | 23 (11.8%) |  | 30 (15.3%) | 0.311 |
| Esophageal and gastric varices (%) | 48 (24.6%) |  | 57 (29.1%) | 0.319 |
| **Active variceal bleeding on endoscopy** | 12 (6.0%) |  | 11 (5.5%) | 0.830 |
| **Endoscopic variceal therapy** | 185 (93.0%) |  | 189 (95.0%) | 0.400 |
| **Surgery or interventional treatment** | 1 (0.5%) |  | 1 (0.5%) | 1.000 |
| **Abbreviations:** MELD, model for end-stage liver disease. | | | | |

| **Supplementary table 13. Subgroup analysis according to the timing of endoscopy defined as the interval from the last presentation of AVB to endoscopy** | | | | | | | | | | | |
| --- | --- | --- | --- | --- | --- | --- | --- | --- | --- | --- | --- |
| ***Subgroup analysis in hematemesis*** | **<12h (N=34)** | **≥12h (N=263)** | **P value** |  | **<24h (N=82)** | **≥24h (N=209)** | **P value** |  | **<48h (N=159)** | **≥48h (N=141)** | **P value** |
| Rate of 5-day failure to control bleeding | 4 (11.8%) | 13 (4.9%) | 0.115 |  | 13 (15.9%) | 7 (3.3%) | ***<0.001*** |  | 17 (10.7%) | 4 (2.8%) | ***0.011*** |
| In-hospital mortality | 4 (11.8%) | 5 (1.9%) | ***0.012*** |  | 5 (6.1%) | 5 (2.4%) | 0.119 |  | 7 (4.4%) | 4 (2.8%) | 0.550 |
| ***Subgroup analysis in non-hematemesis*** | **<12h (N=21)** | **≥12h (N=171)** | **P**  **value** |  | **<24h (N=32)** | **≥24h (N=148)** | **P value** |  | **<48h (N=67)** | **≥48h (N=101)** | **P value** |
| Rate of 5-day failure to control bleeding | 2 (9.5%) | 1 (0.6%) | ***0.032*** |  | 2 (6.3%) | 1 (0.7%) | 0.082 |  | 3 (4.5%) | 1 (1.0%) | 0.303 |
| In-hospital mortality | 1 (4.8%) | 1 (0.6%) | 0.207 |  | 1 (3.1%) | 1 (0.7%) | 0.325 |  | 1 (1.5%) | 1 (1.0%) | 1.000 |
| ***Subgroup analysis in Child-Pugh class A*** | **<12h (N=11)** | **≥12h (N=145)** | **P**  **value** |  | **<24h (N=26)** | **≥24h (N=123)** | **P value** |  | **<48h (N=56)** | **≥48h (N=85)** | **P value** |
| Rate of 5-day failure to control bleeding | 0 (0.0%) | 3 (2.1%) | 1.000 |  | 3 (11.5%) | 1 (0.8%) | ***0.017*** |  | 3 (5.4%) | 1 (1.2%) | 0.301 |
| In-hospital mortality | 1 (9.1%) | 1 (0.7%) | 0.136 |  | 1 (3.8%) | 1 (0.8%) | 0.320 |  | 1 (1.8%) | 1 (1.2%) | 1.000 |
| ***Subgroup analysis in Child-Pugh class B/C*** | **<12h (N=39)** | **≥12h (N=262)** | **P**  **value** |  | **<24h (N=80)** | **≥24h (N=212)** | **P value** |  | **<48h (N=151)** | **≥48h (N=143)** | **P value** |
| Rate of 5-day failure to control bleeding | 5 (12.8%) | 11 (4.2%) | ***0.025*** |  | 11 (13.8%) | 7 (3.3%) | ***0.001*** |  | 16 (10.6%) | 4 (2.8%) | ***0.010*** |
| In-hospital mortality | 4 (10.3%) | 5 (1.9%) | ***0.019*** |  | 5 (6.3%) | 5 (2.4%) | 0.103 |  | 7 (4.6%) | 4 (2.8%) | 0.543 |

| **Supplementary table 14. Subgroup analysis according to the timing of endoscopy defined as the interval from the admission to endoscopy** | | | | | | | | | | | |
| --- | --- | --- | --- | --- | --- | --- | --- | --- | --- | --- | --- |
| ***Subgroup analysis in hematemesis*** | **<12h (N=53)** | **≥12h (N=276)** | **P value** |  | **<24h (N=112)** | **≥24h (N=216)** | **P value** |  | **<48h (N=182)** | **≥48h (N=144)** | **P value** |
| Rate of 5-day failure to control bleeding | 4 (7.5%) | 18 (6.5%) | 0.765 |  | 8 (7.1%) | 15 (6.9%) | 0.947 |  | 11 (6.0%) | 11 (7.6%) | 0.569 |
| In-hospital mortality | 3 (5.7%) | 7 (2.5%) | 0.207 |  | 6 (5.4%) | 5 (2.3%) | 0.147 |  | 7 (3.8%) | 4 (2.8%) | 0.761 |
| ***Subgroup analysis in non-hematemesis*** | **<12h (N=22)** | **≥12h (N=177)** | **P value** |  | **<24h (N=63)** | **≥24h (N=133)** | **P value** |  | **<48h (N=107)** | **≥48h (N=92)** | **P value** |
| Rate of 5-day failure to control bleeding | 1 (4.5%) | 3 (1.7%) | 0.377 |  | 2 (3.2%) | 2 (1.5%) | 0.595 |  | 2 (1.9%) | 2 (2.2%) | 1.000 |
| In-hospital mortality | 1 (4.5%) | 1 (0.6%) | 0.209 |  | 1 (1.6%) | 1 (0.8%) | 0.541 |  | 1 (0.9%) | 1 (1.1%) | 1.000 |
| ***Subgroup analysis in Child-Pugh class A*** | **<12h (N=11)** | **≥12h (N=151)** | **P value** |  | **<24h (N=42)** | **≥24h (N=117)** | **P value** |  | **<48h (N=83)** | **≥48h (N=79)** | **P value** |
| Rate of 5-day failure to control bleeding | 0 (0.0%) | 4 (2.6%) | 1.000 |  | 1 (2.4%) | 3 (2.6%) | 1.000 |  | 1 (1.2%) | 2 (2.5%) | 0.613 |
| In-hospital mortality | 1 (9.1%) | 1 (0.7%) | 0.132 |  | 1 (2.4%) | 1 (0.9%) | 0.460 |  | 1 (1.2%) | 1 (1.3%) | 1.000 |
| ***Subgroup analysis in Child-Pugh class B/C*** | **<12h (N=54)** | **≥12h (N=276)** | **P value** |  | **<24h (N=120)** | **≥24h (N=210)** | **P value** |  | **<48h (N=185)** | **≥48h (N=142)** | **P value** |
| Rate of 5-day failure to control bleeding | 4 (7.4%) | 17 (6.2%) | 0.760 |  | 8 (6.7%) | 14 (6.7%) | 1.000 |  | 11 (5.9%) | 11 (7.7%) | 0.519 |
| In-hospital mortality | 3 (5.6%) | 7 (2.5%) | 0.214 |  | 6 (5.0%) | 5 (2.4%) | 0.202 |  | 7 (3.8%) | 4 (2.8%) | 0.762 |
